# Association between genomic recurrence risk and well-being among breast cancer patients

**DOI:** 10.1186/1471-2407-13-295

**Published:** 2013-06-18

**Authors:** Valesca P Retèl, Catharina GM Groothuis-Oudshoorn, Neil K Aaronson, Noel T Brewer, Emiel JT Rutgers, Wim H van Harten

**Affiliations:** 1 Division of Psychosocial Research and Epidemiology, Netherlands Cancer Institute, Plesmanlaan 121, Amsterdam, CX, 1066, The Netherlands; 2 Governance and Management, MB-HTSR, University of TwenteSchool, PO Box 217, Enschede, AE, 7500, The Netherlands; 3 Gillings School of Global Public Health, University of North Carolina, 325 Rosenau Hall, CB 7440, Chapel Hill, NC, 27599, USA; 4 University of North Carolina, Lineberger Comprehensive Cancer Center, North Carolina, USA; 5 Department of Surgical Oncology, Netherlands Cancer Institute, Plesmanlaan 121, Amsterdam, CX, 1066, The Netherlands

**Keywords:** Personalized medicine, Genomic profile, 70-gene signature, Patient-centered care, Breast cancer, Chemotherapy

## Abstract

**Background:**

Gene expression profiling (GEP) is increasingly used in the rapidly evolving field of personalized medicine. We sought to evaluate the association between GEP-assessed of breast cancer recurrence risk and patients’ well-being.

**Methods:**

Participants were Dutch women from 10 hospitals being treated for early stage breast cancer who were enrolled in the MINDACT trial (Microarray In Node-negative and 1 to 3 positive lymph node Disease may Avoid ChemoTherapy). As part of the trial, they received a disease recurrence risk estimate based on a 70-gene signature and on standard clinical criteria as scored via a modified version of Adjuvant! Online. \Women completed a questionnaire 6–8 weeks after surgery and after their decision regarding adjuvant chemotherapy. The questionnaire assessed perceived understanding, knowledge, risk perception, satisfaction, distress, cancer worry and health-related quality of life (HRQoL), 6–8 weeks after surgery and decision regarding adjuvant chemotherapy.

**Results:**

Women (*n* = 347, response rate 62%) reported high satisfaction with and a good understanding of the GEP information they received. Women with low risk estimates from both the standard and genomic tests reported the lowest distress levels. Distress was higher predominately among patients who had received high genomic risk estimates, who did not receive genomic risk estimates, or who received conflicting estimates based on genomic and clinical criteria. Cancer worry was highest for patients with higher risk perceptions and lower satisfaction. Patients with concordant high-risk profiles and those for whom such profiles were not available reported lower quality of life.

**Conclusion:**

Patients were generally satisfied with the information they received about recurrence risk based on genomic testing. Some types of genomic test results were associated with greater distress levels, but not with cancer worry or HRQoL.

**Trial registration:**

ISRCTN: ISRCTN18543567

## Background

Gene expression profiling, an example of personalized medicine, has moved quickly into clinical care. Breast cancer treatment guidelines that incorporate genomic testing include those from the National Comprehensive Cancer Network (NCCN), the American Society of Clinical Oncology (ASCO), the 2008 Dutch Clinical Guidelines (CBO) and the 2009 St Gallen guidelines [1]. One gene expression test is the 70-gene signature (Mamma-Print™) [2,3] that can accurately distinguish early stage breast tumours at high risk for distant metastasis from low-risk tumours. Several retrospective validation studies have confirmed its prognostic value [4-6], though the effects of receiving results from this test on patient well-being are largely unknown.

A randomized controlled trial, the MINDACT (Microarray In Node-negative and 1 to 3 positive lymph node Disease may Avoid ChemoTherapy; EORTC 10041/BIG 3–04) evaluated prospectively whether the 70-gene signature selects the right patients for adjuvant chemotherapy better than standard clinicopathological criteria [7,8]. This trial enrolled 6700 early stage breast cancer patients throughout Europe, who had their risk of disease recurrence assessed by both standard clinicopathological criteria and the 70-gene signature. Clinicopathological prognostic risk was assessed through a modified version of Adjuvant! Online [9]. Low risk for distant recurrence was defined as >88% chance of 10-years survival for oestrogen receptor (ER)-positive breast cancer and >92% for ER-negative breast cancer. Concordant genomic high (G-high) and clinicopathological high (C-high) risk patients were recommended to undergo adjuvant chemotherapy, and concordant G-low and C-low risk patients were informed that chemotherapy is not recommended. Discordant patients (“C-low/G-high” of “C-high/G-low”) were randomized to treatment-decision making based on the genomic risk assessment or treatment-decision making based on the clinical risk assessment [10].

Since genomic testing is a recent development, relatively few studies have investigated psychosocial issues surrounding these tests. O’Neill et al., in a survey of 139 women who received breast cancer treatment before genomic profiling was available, found a strong interest in genomic testing [11]. Richman et al., in a study of 78 breast cancer patients who had previously received gene expression profiling, reported that many women had an inadequate understanding of gene profiling [12]. In an analysis of data from the same study, Tzeng et al. found that many breast cancer patients preferred a level of shared decision making that was different from what they experienced with their doctors [13]. Lo et al. found that receiving gene expression profiling results lowered patients’ (*n* = 89) anxiety [14]. Both Tzeng et al. and Lo et al. found that patients’ decisions were largely concordant with their gene expression profile results. These studies tended to have small samples, examined the effects of different risk results only minimally, and did not investigate the impact of the combination of gene profiling and clinical risk data in their analyses. Recently, Sulayman et al. reported on the psychosocial and quality of life impact of women receiving an intermediate genomic score [15]. They found that those women who took a passive role in their care reported higher cancer-related distress and cancer worry and lower quality of life than those who took a shared or active role [15].

The primary aims of our study were to evaluate the association between breast cancer patients’ well-being and the results of a gene expression profile on to compare different recurrence risk groups, according to their genomic and standard clinical risk assessments. We expected higher well-being for the concordant “C-low/G-low” risk group (clinical and genomic assessments indicate low risk), lower well-being in patients who did not receive genomic results and lower well being for the discordant “C-low/G-high” risk groups (clinical parameters indicate low risk while genomic test indicates high risk), especially the group who did not receive chemotherapy. In addition, we examined the extent to which women understood the genomic test information received, risk perception, knowledge, and satisfaction with provided information and with the clinical process.

## Methods

### Study sample

Women taking part in the MINDACT-trial from 10 hospitals in the Netherlands were approached to participate in the study. Eligible patients were those with early stage breast cancer (0–3 positive lymph nodes) who were able to read and write in Dutch or English. In addition to the patients enrolled in the MINDACT trial, we also included in our sample women screened for MINDACT trial inclusion, but who ultimately were found ineligible because their genomic results were unavailable (i.e., samples had >3 positive lymph nodes (62%), or insufficient RNA quality or logistical problems (38%)) [10]. The latter occurred primarily during the first period of the study, but decreased thereafter. Clinical tests (C) had two possible results (low or high recurrence risk) and genomic tests (G) had three (low, high or a “not available” (na) recurrence risk). Crossing clinical and genomic results, and accounting for trial assignment of discordant test results, yielded 8 groups: 1) “C-low/G-low”, 2) “C-high/G-high”, 3) “C-low/G-high assigned to no CT”, 4) “C-low/G-high assigned to CT”, 5) “C-high/G-low assigned to no CT”, 6) “C-high/G-low assigned to CT”, 7) “C-low/G-na”, and 8) “C-high/G-na”. This study received review and approvals from the Central Review Board of the University of Maastricht, institutional review boards of the Netherlands Cancer Institute and participating hospitals, the European Organization for Research and Treatment of Cancer Protocol Review Committee, the TRANSBIG and MINDACT Steering Committees and the TRANSBIG Ethical-Legal Committee.

### Procedures

**Figure 1:**
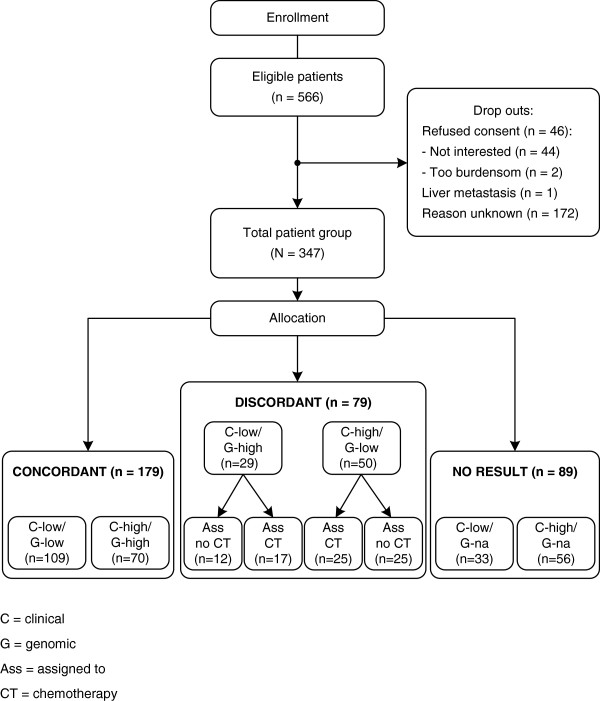
CONSORT diagram.

Patient recruitment began in September, 2008 and continued until the end of August, 2010. Eligible patients received an invitation letter signed by the treating physician, along with the general MINDACT-trial information before surgery. Patients who were enrolled in the MINDACT trial could choose whether to participate in the current study. Patients had surgery to remove their tumors, and then 6–8 weeks later we mailed patients the questionnaire accompanied by an informed consent form. At the time we sent the questionnaires, patients had received assessments of their risk for breast cancer recurrence from the standard clinical markers and the genomic profile and had made a decision regarding adjuvant treatment, but they had not yet started adjuvant treatment. Patients who did not respond to the initial invitation were mailed a reminder two weeks later (See Figure 1). All study materials were in Dutch.

### Measures

During a previous feasibility study, the MicroarRAy PrognoSTics in Breast CancER (RASTER) study [16], we interviewed 27 patients about their personal experiences dealing with the 70-gene signature. Based on these interviews, we constructed a questionnaire and pilot tested it with 77 patients in the same study [17]. A modified version of this questionnaire was used in the current study (See for the total questionnaire Additional file 1).

The questionnaire assessed sociodemographic, clinical and psychosocial variables. The primary study outcome was patient well–being, defined as genomic testing-specific distress (referred to subsequently as distress), cancer-specific worry, and health-related quality of life (HRQoL). Distress was assessed with 10 items adapted from Lynch’s distress scale [18] (α = 0.91). An adapted 7-item version of Lerman’s Cancer Worry Scale [19] (α = 0.89) was used to measure cancer worry. The Breast Cancer Subscale of the Functional Assessment of Cancer Therapy – Breast questionnaire was used to assess HRQoL [20]. We averaged items for each scale to create 3 continuous composite variables.

The questionnaire also measured variables that could have influenced the way patients responded to their test results, as shown in Table 1. We developed 5 items regarding the extent to which women understood the information provided (α = 0.81): 1 item on whether women received both test results at the same or at separate medical visits; 14 items on genomic test knowledge, scored for accuracy and summed to form a “knowledge” index; 1 item on perceived risk of breast cancer recurrence; and 5 items on satisfaction with provided information and process, averaged to form a “satisfaction” scale (α = 0.78).

**Table 1:** Questionnaire measures

**Predictor variables**	**No. of items (response scale)**	α*	**Mean (SD)**	**Item wording (translated from Dutch)**
Perceived understanding	5 items (4-point scale)	0.81	2.89 (0.42)	Did you find the verbal information clear?
				Did you find the written information clear?
				Did you find the information prior to the test clear?
				Did you find the information about handling the results clear?
				Did you find the total information clear, for making a careful decision?
Results received at the same visit	1 item (Yes/no)	NA		Did you receive the test results on one occasion?
Knowledge	14 items (Yes, no, DK)		75% (0.17)	See Table 3
Risk perception	1 item (0-100%)	NA		What do you think is the chance your cancer will come in the coming 10 years?
Satisfaction	5 items (5-point scale)	0.78	2.06 (0.65)	How satisfied were you with the total medical care for breast cancer; with the time you had to wait for the test results; the total information provided; the way the results were conveyed; communication with the medical and nursing staff.
**Outcomes**				
Distress	10 items (4-point scale; a little, some, very, a lot)	0.91	1.99 (0.79)	How did you feel when your doctor told you the [genomic] test results? relieved, glad, disappointed; sad; surprised; confused; upset; insecure; angry; helpless; anxious; guilty; sombre.
Cancer-specific worry	7 items (4-point scale)	0.89	1.79 (0.58)	During the last 4 weeks: how often have you thought about getting cancer again; how often do you worry about getting metastasis; needing chemotherapy again; did this affect your mood?
Quality of life	9 items (5 point scale)	0.63	26.44 (5.11)	Breast cancer subscale of the FACT-B

*Cronbach’s alpha in the present study.

*DK*, don’t know; *NA*, not applicable.

### Data analysis

We imputed values for 61 patients with missing data (17.5%, mostly in the well-being scales), assuming that data are missing at random. The procedure relied on variables in the regression analysis and variables predictive of missing values to create five complete datasets after 100 iterations using fully conditional specification [21]. Multiple imputation is currently regarded as a state-of-the-art technique because it improves accuracy and statistical power relative to other missing data techniques (including list wise deletion). We conducted analyses with each of the five datasets and then pooled results according to Rubin’s rules [22]. As the analysis of the original dataset without imputation yielded the same pattern of findings, and the imputation set is more efficient with more data, we only report results from the imputation dataset in the results.

We assessed baseline differences between groups with Student’s *t*-test, the Mann Whitney-U test, and chi-square tests. We used unadjusted univariate analysis of variance (ANOVA) to evaluate whether the risk groups differed in distress, worry and HRQoL. Block-wise multiple linear regression analysis was carried out to identify variables associated with distress, worry and HRQoL. The first block contained the sociodemographic variables; the second block contained relevant additional factors such as understanding, risk perception, satisfaction, knowledge and receiving both tests on one occasion; the third block contained the genomic test result risk group variables. The “C-low/G-low” group was the reference category. In the regression analysis, the residuals were checked for normality. The R^2^ was calculated according to the formula in Harel, 2009 [21]. In order to maintain the family-wise Type 1 error at 0.05 over the multiple (correlated) tests, we set the critical alpha at a conservative 0.01. We conducted all analyses with SPSS version 17, except Rubin’s rules, for which we used version 18.

## Results

### Study sample

Of 566 patients we invited to participate, 347 returned completed questionnaires (62% response rate; see Figure 1). The characteristics of respondents are shown in Table 2. On average, we received the questionnaires within 3 months (89 days) of sending them. In general, guidelines for Dutch hospitals indicated that, 6 weeks (42 days) after surgery, women should start radiotherapy that takes additional 7 weeks (49 days). Thus, on average respondents would not yet have started (possible) chemotherapy at the time they completed the survey. Concordant risk results were the most common finding: “C-low/G-low” (*n* = 111) and “C-high/G-high” (*n* = 72). Discordant risk results were the least common: “C-low/G-high assigned to no CT” (*n* = 12), “C-low/G-high assigned to CT” (*n* = 18), “C-high/G-low assigned to no CT” (*n* = 25), and “C-high/G-low assigned to CT” (*n* = 25). Genomic results deemed “not available” were also found: “C-low/G-na” (*n* = 33) and “C-high/G-na” (*n* = 56).

**Table 2:** **Characteristics of respondents (*N*** **= 347)**

	***n***	***%***	***Mean (range)***	***SD***
**Age**			55,3 (26–71)	8.8
≤35	10	3		
36-45	37	11		
46-55	119	34		
56-65	139	40		
≥66	42	12		
**Marital status**				
Living as married	274	79		
Not living as married	73	21		
**Had children**				
Yes	274	79		
No	73	21		
**Education**				
Primary school	46	13		
High school	192	55		
College or university	109	32		
**Dutch citizen**				
Yes	325	94		
No	22	6		
**Relatives who underwent CT before**				
Yes	152	44		
No	189	55		
**Recurrence risk group**				
C-low/G-low	109	31		
C-high/G-high	70	20		
C-low/G-high assigned to no CT	12	4		
C-low/G-high assigned to CT	17	5		
C-high/G-low assigned to no CT	25	7		
C-high/G-low assigned to CT	25	7		
C-low/G-na	33	10		
C-high/G-na	56	16		

*Note*. *C*, clinical, *G*, genomic, *CT*, chemotherapy. Analyses that included age treated it as a continuous variable.

### Perceived understanding of the test information

Few women (*n* = 21, 6%) had heard of the 70-gene signature before their diagnosis. Women recalled that they had received information about their risk of metastasis most often in words (*n* = 139, 43%), less commonly in both words and numbers (n = 100, 31%), and rarely in numbers only (*n* = 25, 8%); the remaining patients did not respond to this question. In general, women found the information they received to be understandable: the written information was perceived as clear by 87% of the women, verbal information by 87%, information prior to the test results by 85%, information about adjuvant treatment by 82%, and information necessary to make a careful decision by 83%. Twenty-seven percent of the women received both test results at the same medical visit (on average two weeks after surgery); 71% received them on two successive occasions (clinical assessment on average one week after surgery, and GEP results on average two weeks after surgery); and for the remaining 2% this was unknown.

### Knowledge and perceived risk

Knowledge about genomic recurrence risk testing was relatively high (mean correct answers, across 14 items = 75%) (Tables 3 and 4). Two questions that elicited substantially more “I don’t know” responses were “The result of the genomic profile is always correct” (43% don’t know); “For a breast tumour with a high risk genomic profile, the chance of metastasis in the next 10 years is 50%” (53% don’t know). The three questions with the most incorrect answers were: “A high genomic profile indicates that a patient will need to have her lymph nodes removed” (25%); “The genomic profile indicates the chance of metastasis” (23%); and “For a breast tumour that the genomic profile indicates as high risk, the chance of metastasis in the next 10 years is 50%” (20%). Women with relatives who previously underwent chemotherapy answered more questions correctly (on average 78% versus 73% correct answers *p* = 0.006). On average, patients perceived their risk of recurrence to be 26.6%.

**Table 3:** **Knowledge (*N*** **= 347)**

	**Correct**	**Incorrect**	**I don’t know**
	**(%)**	**(%),**	**(%)**
**Correct answer was “true”**			
The GP is done on tumour tissue from the breast removed by surgery.	97	1	2
The GP is based on the genes of the breast tumour.	90	4	6
The GP help some women avoid having unneeded chemotherapy.	90	4	6
A patient with a high risk tumour will be recommended chemotherapy.	86	5	9
The GP gives the chance of metastasis.	67	23	10
For a high risk tumour, the chance of metastasis in the next 10 years is over 50%.	27	21	52
**Correct answer was “false”**			
The GP is done before surgery that removes the tumour.	88	6	6
Only the GP is used by the doctor to recommend chemo.	88	6	6
A GP tells whether other women in the family have higher risk of breast cancer.	78	11	11
The GP tells whether cancer cells have spread to the lymph nodes.	74	18	8
The GP can help women to decide about the sort of breast cancer surgery to undergo.	70	17	13
The GP looks at all genes in a patient’s body.	69	14	17
A high risk GP indicates that a patient will need to have lymph nodes removed.	62	25	13
The GP is always correct.	38	19	43

*Note*. *GP*, genomic profile.

**Table 4:** Mean satisfaction, perceived understanding, knowledge and risk perception

	**Satisfaction**	**Perceived understanding**	**Knowledge**	**Risk perception**
	**(95% CI)**	**(95% CI)**	**(95% CI)**	**(95% CI)**
C-low/G-low	2.02 (1.92-2.13)	2.91 (2.84-2.98)	0.77 (0.74-0.80)	22.38 (18.28-26.48)
C-high/G-high	2.08 (1.92-2.24)	2.94 (2.84-3.04)	0.78 (0.73-0.82)	31.60 (24.78-38.42)
C-low/G-high (ass. no CT)	2.23 (1.70-2.77)	2.65 (2.31-2.99)	0.73 (0.59-0.86)	35.91 (20.17-51.65)
C-low/G-high (ass. CT)	2.15 (1.82-2.49)	2.88 (2.65-3.12)	0.77 (0.70-0.84)	30.31 (17.91-42.71)
C-high/G-low ass. no CT	2.08 (1.78-2.38)	2.88 (2.69-3.08)	0.78 (0.75-0.79)	27.09 (15.43-38.74)
C-high/G-low (ass.CT)	1.97 (1.63-2.31)	2.94 (2.78-3.11)	0.76 (0.69-0.83)	22.05 (12.37-31.73)
Overall	2.06 (1.98-2.14)	2.90 (2.85-2.96)	0.77 (0.75-0.79)	26.50 (23.43-29.56)
*p*	0.831	0.353	0.952	0.115

*Note*. *n* = 347. The reference group in analyses was C-low/G-low; *p* value is for omnibus test. *C*, clinical, *G*, genomic, ass. = randomly assigned to receive, *CT*, chemotherapy.

Satisfaction scale: “very satisfied” (coded as 1), ”satisfied” (2), ”neutral” (3), ”unsatisfied” (4), ”very unsatisfied” (5).

Perceived understanding scale : “not at all clear” (1), ”somewhat clear” (2), ”moderately clear” (3), ”completely clear” (4).

Knowledge scale : percentage of correctly answered questions.

Risk perception scale: “no risk of developing metastasis within 10 years” (0),“100% risk” (100).

### Satisfaction

Almost all women (97%) were satisfied with their experience from diagnosis up to the time that the questionnaire was completed. Similarly, 94% expressed overall satisfaction with the information received. Twenty-eight percent of the patients were unsatisfied with the waiting time for the results. Based on self-report data, 6% received results within 1 week of surgery, 23% within 2 weeks, 29% within 3 weeks, 23% within 4 weeks, and 18% after more than 4 weeks. Nine percent of the patients expressed dissatisfaction with the way in which doctors conveyed the results (Table 4).

### Distress

**Figure 2:**
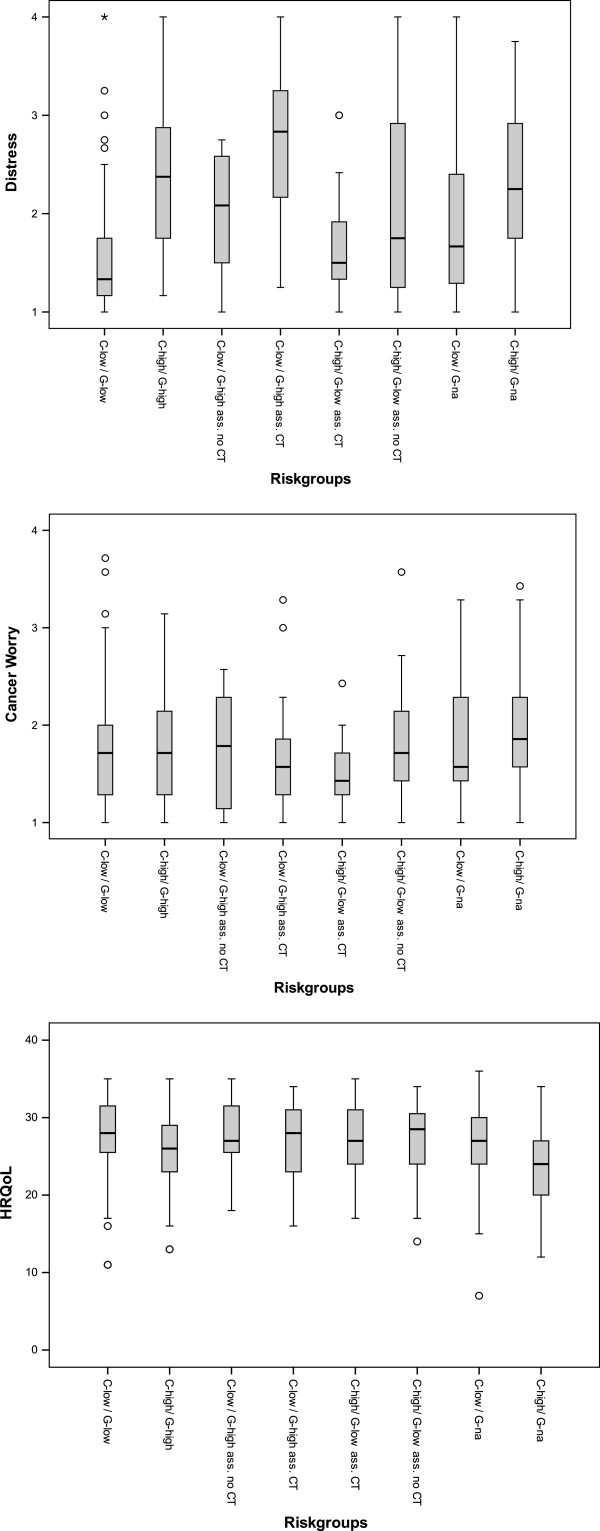
**Unadjusted analysis for Distress, Cancer worry and Health-related quality of life (*N*** **= 347).** Box plots show median line, quartiles and outliers. C= clinical, G= genomic, Ass= assigned to, CT= chemotherapy.

In the unadjusted (univariate) analysis, distress was different among the risk groups (*p* = 0.017) (Figure 2, Table 5). In the adjusted (regression) analysis, risk group remained associated with distress levels after controlling for sociodemographic, information/knowledge, and risk perception variables (Table 6). The group “C-low/G-low” (reference) reported the lowest distress, not different from the group “C-high/G-low assigned to chemotherapy” (*p* = 0.18). Associated with higher distress compared to the reference group were the groups: genomic profile not available (*p* = 0.002 and *p* < 0.001), “C-high/G-high” (*p* = 0.01) and the discordant groups “C-low/G-high assigned to CT” (*p* < 0.001) and “C-high/G-low assigned to no CT” (*p* < 0.001) (Table 6).

**Table 5:** **Accompanying Figure**2

	**Distress**	**Worry**	**Quality of life**
	***Mean (95% CI)***	***Mean (95% CI)***	***Mean (95% CI)***
C-low/G-low	1.52 (1.42 to 1.62)	1.73 (1.63 to 1.84)	28.0 (27.1 to 28.9)
C-high/G-high	2.39^*^ (2.21 to 2.56)	1.82 (1.69 to 1.95)	25.6^*^ (24.4 to 26.9)
C-low/G-high (assigned to no CT)	2.03^*^ (1.64 to 2.41)	1.77 (1.41 to 2.14)	28.0 (24.9 to 31.1)
C-low/G-high (assigned to CT)	2.71^*^ (2.35 to 3.07)	1.72 (1.40 to 2.05)	26.3 (23.6 to 29.0)
C-high/G-low (assigned to no CT)	2.11^*^ (1.70 to 2.52)	1.85 (1.61 to 2.10)	26.9 (24.6 to 29.1)
C-high/G-low (assigned to CT)	1.68 (1.45 to 1.91)	1.49 (1.34 to 1.65)	26.9 (25.1 to 28.8)
C-low/G-na	1.95^*^ (1.58 to 2.32)	1.86 (1.61 to 2.10)	26.0 (23.9 to 28.2)
C-high/G-na	2.31^*^ (2.11 to 2.51)	1.95 (1.80 to 2.11)	23.8^*^ (22.5 to 25.2)

*Note*. The reference group in analyses was C-low/G-low. *C*, clinical, G = genomic, *CT*, chemotherapy, *na*, result not available. *n* = 347.

*p < 0.01.

**Table 6:** Correlates of distress, worry and quality of life in adjusted analyses

	***Distress***			***Worry***			***Quality of life***		
	***b***	***(se)***	***p***	***b***	***(se)***	***p***	***b***	***(se)***	***p***
**Block 1**									
Age	−0.001	(0.005)	0.821	−0.042	(0.021)	0.049	0.122	(0.034)	0.000*
Married	−0.046	(0.099)	0.645	0.647	(0.422)	0.125	1.165	(0.680)	0.087
Had children	0.124	(0.097)	0.203	−0.089	(0.442)	0.841	−1.566	(0.689)	0.023
Primary school (vs. High school)	−0.018	(0.126)	0.885	−0.307	(0.583)	0.598	−1.864	(0.869)	0.032
College (vs. High school)	−0.185	(0.088)	0.036	−0.125	(0.407)	0.759	0.243	(0.639)	0.703
**Block 2**									
Understanding	−0.199	(0.113)	0.079	−0.963	(0.497)	0.052	−0.119	(0.756)	0.875
Results received in 1 visit	−0.016	(0.085)	0.849	−0.224	(0.378)	0.554	0.187	(0.593)	0.753
Knowledge	−0.021	(0.014)	0.137	−0.127	(0.056)	0.023	0.109	(0.086)	0.203
Risk perception	0.003	(0.002)	0.128	0.049	(0.008)	0.000*	−0.043	(0.012)	0.000*
Satisfaction	0.151	(0.071)	0.032	0.852	(0.323)	0.008*	−0.517	(0.481)	0.282
**Block 3**									
C-high/G-high	0.877	(0.105)	0.000*	0.107	(0.494)	0.827	−1.908	(0.769)	0.013
C-low/G-high (assigned to no CT)	0.423	(0.209)	0.043	−1.085	(0.962)	0.259	0.793	(1.453)	0.585
C-low/G-high (assigned to CT)	1.115	(0.178)	0.000*	−0.610	(0.816)	0.455	−1.407	(1.235)	0.254
C-high/G-low (assigned to no CT)	0.611	(0.153)	0.000*	0.435	(0.706)	0.538	−0.671	(1.099)	0.541
C-high/G-low (assigned to CT)	0.221	(0.163)	0.175	−1.230	(0.705)	0.081	−1.131	(1.081)	0.296
C-low/G-na	0.488	(0.152)	0.002*	0.427	(0.651)	0.512	−1.752	(0.984)	0.075
C-high/G-na	0.710	0.126)	0.000*	0.842	(0.527)	0.111	−3.816	(0.824)	0.000*

*Note*. *b* = unstandardized pooled regression coefficient, se = standard error, C = clinical, G = genomic, CT = chemotherapy, na = result not available. Reference group for recurrence risk groups was C-low/G-low.

Distress: Block 1 R^2^ = 0.040 Block 2 R^2^ = 0.147; Block 3: R^2^ = 0.385.

Worry: Block 1 R^2^ = 0.039 Block 2 R^2^ = 0.228; Block 3: R^2^ = 0.251.

Quality of Life: Block 1 R^2^ = 0.094 Block 2 R^2^ = 0.156; Block 3 R^2^ = 0.219.

*p < .01.

### Worry

In the unadjusted analysis, the 8 risk groups had similar levels of worry (*p* = 0.234) (Figure 2). In the adjusted analysis, corrected for demographic factors, higher levels of worry were observed among women who expressed lower satisfaction (*p* < 0.001) and among women with higher perceived risk (*p* < 0.001) (See Table 6).

### HRQoL

In the unadjusted analysis, HRQoL was different among the risk groups (*p* = 0.024) (Figure 2). In the adjusted analysis, older age was associated with better HRQoL (*p* < 0.001), while higher risk perception was associated with lower HRQoL (*p* < 0.001). After controlling for demographic and process factors, only the “C-high/G-na” (p < 0.001) risk group reported lower HRQoL compared to the reference group “C-low/G-low” (See Table 6).

## Discussion

In general, women indicated that the information they received regarding the test results was clear and satisfactory and resulted in a good understanding of the genomic profile and its consequences. As expected, we found the least distress among patients with a low recurrence risk according to standard and genomic indicators. Most other groups had statistically significant or marginally higher distress except for patients with high risk according to clinical indicators, low recurrence risk according to the genomic profile and assigned to chemotherapy. Our expectation that higher distress, more worry and lower HRQoL would be observed among the discordant “C-low/G-high” risk groups was not confirmed. Rather, higher distress levels compared to the reference group were observed for the “C-low/G-high assigned to chemotherapy” and “C-high/G-low not assigned to chemotherapy”. Higher distress among patients with uniformly high risk results or with uncertain genomic results makes intuitive sense. Patients with discordant results appear to have had a lingering sense of unease. They may have derived comfort from receiving chemotherapy together with a low genomic risk result. However, either one on its own appears to have been insufficient to ameliorate women’s distress. In the future, the genomic risk profile results may become incorporated into clinical guidelines, such that patients would receive only one test outcome, which could help to reduce uncertainty and distress.

Although we expected high correlations among the three study outcomes (distress, worry and quality of life), they were only moderately correlated. Furthermore, we found distinct correlates of each. Distress levels tended to vary primarily as a function of risk group, whereas worry was associated with risk perception and satisfaction. Lower quality of life was associated with younger age, higher perceived risk, and the risk group with “C-high/G-na” result. These differences may be due, in part, to the varying focus of these three measures. The distress scale assesses primarily distress related to the genomic results, while the worry scale assesses breast cancer-related worry, and the quality of life measure taps into both general and breast cancer-related issues.

Strengths of the study include its larger sample compared to previous studies, its multicenter and prospective research design, and the use of standardized measures to assess psychosocial outcomes. The distribution of patients across the subgroups and the general characteristics of the sample were comparable to those of the predefined pilot phase of the MINDACT trial [10].

The study also had several limitations. First, while we were able to form 8 groups on the basis of clinical and genomic risk status and treatment decision, the groups with discordant risk estimates were relatively small, and thus may have limited the power of the study to detect group differences. This may explain, in part, why we did not confirm the hypothesis that the “C-low/G-high assigned to no chemotherapy” risk group would have higher distress. The “C-low/G-low” group was chosen as the reference category, because we were not able to compare the risk groups with patients who did not receive or were not prepared to receive a genomic profile. Furthermore, the results of the multivariate analysis did not change by using only the MINDACT trial eligible respondents (leaving out the no-genomic results group). Second, the response rate in this study was moderate (62%), although we would note that it is in line with that observed in other randomized EORTC trials [23,24]. Third, we could not distinguish the difference between the genomic or clinical low group separately, because the patients in the clinical trial were randomized directly after the results were known. So, the effects of the various risk profiles on well-being could not been examined independent of the effect of the adjuvant treatment advice. Although our study sample included both women who had and had not been randomized into the MINDACT trial, including these patients did not appear to have changed our results. When we performed the same analyses for the randomized group only, the results were in line with those based on the combined sample (data not shown). Finally, it could be that some patients may have completed the survey after starting chemotherapy, which means that there could be a small confusion bias between the impact of the result and undergoing chemotherapy.

## Conclusion

Our results indicate that the current system of providing genomic test results as in the MINDACT trial works well. Women found the information they received was clear and satisfactory and helped them understand the genomic profile and its consequences. While these findings are encouraging, clinicians should be aware that genomic test results may be associated with patients’ wellbeing. Especially for patients with high recurrence risk or discordant risk test results, it may be advisable to offer additional psychological counselling, as such counselling can reduce distress associated with the results of genetic tests [25].

## Abbreviations

GEP: Gene expression profiling; MINDACT trial: “Microarray In Node-negative and 1 to 3 positive lymph node Disease may Avoid ChemoTherapy” trial; HRQoL: Health-related quality of life; NCCN: National Comprehensive Cancer Network; ASCO: American Society of Clinical Oncology; CBO: Dutch clinical guidelines; ER: Estrogen receptor; C-low: Clinical parameters indicated low recurrence risk; C-high: Clinical parameters indicated high recurrence risk; G-low: Genomic test indicated low recurrence risk; G-high: Genomic test indicated high recurrence risk; Na: Not available; ANOVA: Analysis of variance.

## Competing interests

W. H. van Harten is a non-remunerated, non-stake holding member of the supervisory board of Agendia BV. All other authors declared no conflicts of interest.

## Authors’ contributions

VR carried out the acquisition of the data, performed the analyses and drafted the manuscript. CGO made substantial contribution to the analysis and co-drafted the manuscript. NB and NA have made substantial contributions to the critical revision of the questionnaire, analyses and manuscript. ER made acquisition of the data possible and was principal investigator and spokesperson of the MINDACT trial. WvH participated in its conception and design and coordination and helped to draft the manuscript. All authors read and approved the final manuscript.

## Pre-publication history

The pre-publication history for this paper can be accessed here:

http://www.biomedcentral.com/1471-2407/13/295/prepub

## Supplementary Material

Additional file 1 Patient questionnaire research on the experience of the MammaPrintTM(70-gene prognosis profile, microarray test). Click here for file
