# Supplementary material for: Association between genomic recurrence risk and well-being among breast cancer patients
Source: BMC Cancer. 2013 Jun 18;13:295. doi: 10.1186/1471-2407-13-295 (PMC3689597; doi:10.1186/1471-2407-13-295)
Supplement: Additional file 1 — Patient questionnaire research on the experience of the MammaPrintTM(70-gene prognosis profile, microarray test). [file 1471-2407-13-295-S1.doc]

**Patient Questionnaire**

**Research on the experience of the MammaPrintTM**

**(70-gene prognosis profile, microarray test)**

Today’s date: ___-___-_____

Hospital: _____________________

**INSTRUCTIONS**

In the process to determine your treatment plan, a breast cancer test (gene expression profile/70-gene prognosis signature) is performed. In this questionnaire, we will call it the “MammaPrint^TM^”. *It is possible that you have not received the Mammaprint eventually, however we would like you to fill in the questionnaire as well.*

We would like to have your insight and opinion about the information about the microarray test and accompanying procedures and what it meant for you to receive the test results.

Take your time to fill in the questionnaire. It is important to answer the questions as carefully as you can. If you are unsure of an answer, please give us your best approximation.

Filling out this questionnaire will take about 30 min.

**Thank you very much for your time and effort!**

**GENERAL INFORMATION**

We would like to ask you some general personal information:

1. What is your date of birth? ___-___-______(month-day-year)
2. What is your marital status?
   - Married or living as married
   - Divorced
   - Widowed
   - Separated
   - Never married
3. Do you have children?
   - No
   - Yes, I have ______ children
4. How would you describe your primary race or ethnicity?

- - African American or black (not of Hispanic origin)
  - White or Caucasian (not of Hispanic origin)
  - Hispanic
  - American Indian or Alaskan Native
  - Asian or Pacific Islander
  - Other, please specify ____________________________________

1. What is the highest level of education you have completed?
   - Less than high school
   - Some high school
   - High school graduate
   - Some college
   - College graduate
   - Post graduate
   - Other, namely ____________________________________

**GENERAL INFORMATION**

1. W hat is or was your profession before the diagnosis? ________________________________
2. Before your breast cancer diagnosis, did you work for pay?
   - No
   - Yes, full time
   - Yes, part time, ________%
3. Roughly what is your total house income per year in dollars (what you have to live on)?

- Less than $20,000
- $20,001 to $60,000
- $60,001 to $100,000
- More than $100,000
- Other ______________________
- I don’t know

1. After your diagnosis, have you incurred extra costs which you must pay for yourself?

Medication:

- No
- Yes, about $ __________________________________________

Travel costs:

- No
- Yes, about $__________________________________________

Home care:

- No
- Yes, about $__________________________________________

1. Did you know prior to receiving your diagnosis of the existence of the microarray test?

- Yes
- No

**THE MICROARRAY TEST AND CHEMOTHERAPY**

You will read now a number of statements; would you check off whether or not you consider the statement to be true or false? Don’t worry if you’re not sure, this is only so that we may judge the quality of our information material.

|  | True | False | Don’t know |
| --- | --- | --- | --- |
| 1. The microarray test looks at all genes in a patient’s body. | 🞎 | 🞎 | 🞎 |
| 1. The microarray test result tells whether cancer cells have spread to the lymph nodes (to part of the body other than the breasts). | 🞎 | 🞎 | 🞎 |
| 1. The microarray test result gives the chance of metastasis (cancer coming back in parts of the body other than the breast). | 🞎 | 🞎 | 🞎 |
| 1. An unfavorable (or high risk) microarray test result indicates that a patient will need to have lymph nodes removed. | 🞎 | 🞎 | 🞎 |
| 1. The microarray test is done on tumor tissue from the breast that is removed when the woman has surgery. | 🞎 | 🞎 | 🞎 |
| 1. The microarray test result is based on the genes of the breast tumor. | 🞎 | 🞎 | 🞎 |
| 1. The result of the microarray test is always correct. | 🞎 | 🞎 | 🞎 |
| 1. For a breast tumor that the microarray test result is unfavorable or high risk, the chance of metastasis in the next 10 years is over 50%. | 🞎 | 🞎 | 🞎 |
| 1. Only the microarray test result is used by the doctor to recommend chemotherapy. | 🞎 | 🞎 | 🞎 |
| 1. The microarray test result tells whether other women in a patient’s family have a higher chance of breast cancer. | 🞎 | 🞎 | 🞎 |
| 1. The microarray test is done before the surgery that removes the breast tumor. | 🞎 | 🞎 | 🞎 |
| 1. The result of the microarray test help some women avoid having unneeded chemotherapy. | 🞎 | 🞎 | 🞎 |
| 1. The microarray test can help women to decide about the sort of breast cancer surgery to undergo. | 🞎 | 🞎 | 🞎 |
| 1. A patient with an ‘unfavorable’’ (or high risk) tumor will be recommended to get chemotherapy. | 🞎 | 🞎 | 🞎 |
| 1. Chemotherapy is a treatment for cancer using medicine. | 🞎 | 🞎 | 🞎 |
| 1. Chemotherapy could lower the chance of metastasis. | 🞎 | 🞎 | 🞎 |
| 1. Hormonal therapy could lower the chance of metastasis. | 🞎 | 🞎 | 🞎 |
| 1. Other medicines can change the effectiveness of chemotherapy. | 🞎 | 🞎 | 🞎 |
| 1. Chemotherapy is used only if it will completely cure the cancer. | 🞎 | 🞎 | 🞎 |
| 1. Chemotherapy can cause hair loss. | 🞎 | 🞎 | 🞎 |
| 1. Breast cancer cells can spread via the lymph nodes. | 🞎 | 🞎 | 🞎 |

**INFORMATION CONCERNING THE MICROARRAY TEST**

Questions 32-37 deal primarily with opinions about the information of the microarray test. Please indicate how clear you found the information.

|  | Not at all clear | Somewhat clear | Moderately clear | Completely clear |
| --- | --- | --- | --- | --- |
| 1. I found the written information… | 🞎 | 🞎 | 🞎 | 🞎 |
| 1. I found the verbal information… | 🞎 | 🞎 | 🞎 | 🞎 |
| 1. I found the information prior to the test… | 🞎 | 🞎 | 🞎 | 🞎 |
| 1. I found the information around handing over the results… | 🞎 | 🞎 | 🞎 | 🞎 |
| 1. I found the information around the possible future treatment… | 🞎 | 🞎 | 🞎 | 🞎 |
| 1. For making a carefully considered decision about future treatment, I found the total information… | 🞎 | 🞎 | 🞎 | 🞎 |

1. Please tell us how your doctor explained your chance of metastasis. Check all that apply.

- Using words. What were they?___________________________________
- Using percentages. What were they?_______________________________
- Both words and percentages. What were they? _______________________
- Other, namely: __________________________________________

1. Do you have the idea that you have had conflicting test information?

- No
- Yes
- Partly

Questions 40 – 43 ask about a number of possible ways to present the chance of metastasis. Your physician likely used one of these. Please indicate how easy (not easy, somewhat, moderately or very easy) each one is to understand.

|  | Not easy | Somewhat easy | Moderately easy | Very easy |
| --- | --- | --- | --- | --- |
| 1. The explanation worded “your chance of metastasis is low” to be… | 🞎 | 🞎 | 🞎 | 🞎 |
| 1. The use of percentages “your chance of metastasis is 6%” to be… | 🞎 | 🞎 | 🞎 | 🞎 |
| 1. The combination, “You have about a 6% chance of metastasis, which is low risk” to be… | 🞎 | 🞎 | 🞎 | 🞎 |
| 1. The use of a table or figure to show what chance of metastasis is to be… | 🞎 | 🞎 | 🞎 | 🞎 |

**THE RESULTS OF THE PATHOLOGY TEST AND MICROARRAY TEST**

There are two ways to know your chance for metastasis. Pathology tests use standard clinical information such as size of your tumor and whether it spread to your lymph nodes. A new way used is the microarray test. The questions below go over the results of the pathology test and the microarray test:

1. Did you receive the results of the pathology test at the same time as the microarray test results? (in one meeting)

- Yes
- No
- I don’t remember

1. Who gave you the results of the pathology tests?

- Internist/Oncologist
- Surgeon
- Nurse practitioner
- Mamma care nurse
- Radiotherapist
- I don’t remember
- Other, namely:_______________________________________________

1. What was the result of the pathology test?

- Low risk
- High risk
- I don’t know
- Other, namely:___________________________________

1. Who gave you the result of the microarray test?

- Internist/Oncologist
- Surgeon
- Nurse practitioner
- Mamma care nurse
- Radiotherapist
- Not applicable, I did not receive the result
- Other, namely:_______________________________________________

1. What was the result of the microarray test?

- Low risk
- High risk
- Not applicable, I did not receive the result
- Other, namely:___________________________________

1. How many days after the breast surgery did you have to wait for the results of the microarray test?

­­­­­­­­­­­­­­­­­_____days after the breast surgery

**TREATMENT AFTER SURGERY**

1. Before you received the microarray test results, which treatment did you think would be the best for you? This does not include radiation treatment.

- Chemotherapy
- Hormone therapy
- Chemotherapy and hormone therapy
- Watchful waiting
- Return only for routine check-up
- I hadn’t thought about it
- Other, namely: ­­­_______________________________________________

1. Which advice for follow-up treatment has the physician ultimately decided upon (aside from radiation treatment)?

- Chemotherapy
- Hormone therapy
- Chemotherapy and hormone therapy
- Watchful waiting
- Return only for routine check-up
- Other, namely: ­­­_______________________________________________

1. Did you eventually follow your doctor’s advice?

- Yes
- No

The next questions are about your motivation to whether or not to agree with your doctor’s advice regarding undergoing chemotherapy:

| I followed the advice of my doctor, because: | Agree completely | Agree | Neither agree, nor disagree | Disagree | Completely disagree | Not applicable/haven’t thought about it |
| --- | --- | --- | --- | --- | --- | --- |
| 1. The doctor is the expert. | 🞎 | 🞎 | 🞎 | 🞎 | 🞎 | 🞎 |
| 1. My family thinks I should. | 🞎 | 🞎 | 🞎 | 🞎 | 🞎 | 🞎 |
| 1. I believe I should avail myself of every chance to get better. | 🞎 | 🞎 | 🞎 | 🞎 | 🞎 | 🞎 |
| 1. My doctor had good reasons for the recommended treatment plan that eliminated my concerns. | 🞎 | 🞎 | 🞎 | 🞎 | 🞎 | 🞎 |
| I **didn’t** follow the advice of my doctor, because: |  | | | | | |
| 1. I thought the odds of winning too low to justify such a difficult treatment. | 🞎 | 🞎 | 🞎 | 🞎 | 🞎 | 🞎 |
| 1. In my opinion, I am recovered enough. | 🞎 | 🞎 | 🞎 | 🞎 | 🞎 | 🞎 |
| 1. I found the hormone therapy difficult enough. | 🞎 | 🞎 | 🞎 | 🞎 | 🞎 | 🞎 |
| 1. Other reason: |  | | | | | |

**EMOTIONS OR FEELINGS AFTER RECEIVING THE MICROARRAY TEST RESULT**

1. Please tell us how you felt after your doctor told you the result of the microarray test. How much did you feel:

|  |  | Not at all | A little | A lot | Very much |
| --- | --- | --- | --- | --- | --- |
| a | Relieved | 🞎 | 🞎 | 🞎 | 🞎 |
| b | Upset | 🞎 | 🞎 | 🞎 | 🞎 |
| c | Surprised | 🞎 | 🞎 | 🞎 | 🞎 |
| d | Sad | 🞎 | 🞎 | 🞎 | 🞎 |
| e | Confused | 🞎 | 🞎 | 🞎 | 🞎 |
| f | Disappointed | 🞎 | 🞎 | 🞎 | 🞎 |
| g | Glad | 🞎 | 🞎 | 🞎 | 🞎 |
| h | Somber | 🞎 | 🞎 | 🞎 | 🞎 |
| i | Insecure | 🞎 | 🞎 | 🞎 | 🞎 |
| j | Angry | 🞎 | 🞎 | 🞎 | 🞎 |
| k | Guilty | 🞎 | 🞎 | 🞎 | 🞎 |
| l | Anxious | 🞎 | 🞎 | 🞎 | 🞎 |
| m | Helpless | 🞎 | 🞎 | 🞎 | 🞎 |

1. Do you have relatives who have had chemotherapy once?

- Yes
- No

**WORRIES ABOUT CANCER**

The next questions are about possible concerns you may have about cancer.

1. Setting aside what your doctor thinks, what do YOU think is the chance your cancer will come back (metastasis)? Place a mark on the line to indicate your answer.

0%

100%

60%

20%

80%

40%

1. How often have you thought about your chances of getting breast cancer again?

| Rarely or never | Sometimes | Often | All the time |
| --- | --- | --- | --- |
|  |  |  |  |

1. Have these thoughts about your chances of getting cancer again affected your mood?

| Rarely or never | Sometimes | Often | All the time |
| --- | --- | --- | --- |
|  |  |  |  |

1. Have these thoughts about your chances of getting cancer again interfered with your ability to do daily activities?

| Rarely or never | Sometimes | Often | All the time |
| --- | --- | --- | --- |
|  |  |  |  |

1. How concerned are you about the possibility of getting cancer again one day?

| Not at all | Somewhat | Moderately concerned | Very concerned |
| --- | --- | --- | --- |
|  |  |  |  |

1. How often do you worry about developing cancer again?

| Rarely or never | Sometimes | Often | All the time |
| --- | --- | --- | --- |
|  |  |  |  |

1. How much of a problem is this worry?

| Not at all | Somewhat | Moderately concerned | Very concerned |
| --- | --- | --- | --- |
|  |  |  |  |

1. How worried are you about the possibility that you will need chemotherapy again?

| Rarely or never | Sometimes | Often | All the time |
| --- | --- | --- | --- |
|  |  |  |  |

**DECISION MAKING**

The next questions are about decisions you made recently.

|  | Agree completely | Agree | Neither agree, nor disagree | Disagree | Completely disagree | Not applicable/haven’t thought about it |
| --- | --- | --- | --- | --- | --- | --- |
| 1. If I had to decide again today, I would still agree to have the microarray test done. | 🞎 | 🞎 | 🞎 | 🞎 | 🞎 | 🞎 |
| 1. I would have made the same decision again about whether or not to have chemotherapy. | 🞎 | 🞎 | 🞎 | 🞎 | 🞎 | 🞎 |
| 1. I have the feeling that I am steered to undergo chemotherapy | 🞎 | 🞎 | 🞎 | 🞎 | 🞎 | 🞎 |
| 1. I would recommend other women in my situation to have the microarray test done. | 🞎 | 🞎 | 🞎 | 🞎 | 🞎 | 🞎 |

1. Please indicate which of the following statements best describes how you want your treatment decision to be made. Please choose only one answer.

- I prefer to make the final selection about which treatment I will receive.
- I prefer to make the final selection of my treatment after seriously considering my doctor’s opinion.
- I prefer that my doctor and I share responsibility for deciding which treatment is best for me.
- I prefer that my doctor makes the final decision about which treatment will be used, but seriously considers my opinion.
- I prefer to leave all decisions regarding my treatment to my doctor.

**SATISFACTION**

Please describe your satisfaction with each of the following:

|  | Very satisfied | satisfied | Neutral | Un-satisfied | Very un-satisfied |
| --- | --- | --- | --- | --- | --- |
| 1. Your total medical care for breast cancer. | 🞎 | 🞎 | 🞎 | 🞎 | 🞎 |
| 1. The time you waited to find out the results of the surgery. | 🞎 | 🞎 | 🞎 | 🞎 | 🞎 |
| 1. Over the total information provided. | 🞎 | 🞎 | 🞎 | 🞎 | 🞎 |
| 1. The way my results were conveyed. | 🞎 | 🞎 | 🞎 | 🞎 | 🞎 |
| 1. Over the communication with the medical or nursing staff. | 🞎 | 🞎 | 🞎 | 🞎 | 🞎 |
| 1. This questionnaire. | 🞎 | 🞎 | 🞎 | 🞎 | 🞎 |

Could you explain why you were or were not satisfied?

__________________________________________________________________________________________________________________________________________________________________________________________________________________

Was there something else you were or were not satisfied about? __________________________________________________________________________________________________________________________________________________________________________________________________________________

**QUALITY OF LIFE**

Below is a list of statements that other people with your illness have said are important. **By circling one (1) number per line, please indicate how true each statement has been for you during the past 7 days.**

|  | PHYSICAL WELL-BEING | | **Not at all** | **A little bit** | **Some-what** | **Quite**  **a bit** | **Very much** |
| --- | --- | --- | --- | --- | --- | --- | --- |
|  |  |  |  |  |  |  |  |
|  |  |  |  |  |  |  |  |
|  |  |  |  |  |  |  |  |
| GP1 | I have a lack of energy………………………………… | | 🞎 | 🞎 | 🞎 | 🞎 | 🞎 |
| GP2 | I have nausea 0 | | 🞎 | 🞎 | 🞎 | 🞎 | 🞎 |
| GP3 | Because of my physical condition, I have trouble  meeting the needs of my family 0 | | 🞎 | 🞎 | 🞎 | 🞎 | 🞎 |
| GP4 | I have pain 0 | | 🞎 | 🞎 | 🞎 | 🞎 | 🞎 |
| GP5 | I am bothered by side effects of treatment 0 | | 🞎 | 🞎 | 🞎 | 🞎 | 🞎 |
| GP6 | I feel ill 0 | | 🞎 | 🞎 | 🞎 | 🞎 | 🞎 |
| GP7 | I am forced to spend time in bed………………………. | | 🞎 | 🞎 | 🞎 | 🞎 | 🞎 |
|  | | | | | | | |
|  | SOCIAL/FAMILY WELL-BEING | | **Not at all** | **A little bit** | **Some-what** | **Quite**  **a bit** | **Very much** |
|  |  |  |  |  |  |  |  |
|  |  |  |  |  |  |  |  |
|  |  |  |  |  |  |  |  |
| GS1 | I feel close to my friends 0 | | 🞎 | 🞎 | 🞎 | 🞎 | 🞎 |
| GS2 | I get emotional support from my family 0 | | 🞎 | 🞎 | 🞎 | 🞎 | 🞎 |
| GS3 | I get support from my friends 0 | | 🞎 | 🞎 | 🞎 | 🞎 | 🞎 |
| GS4 | My family has accepted my illness 0 | | 🞎 | 🞎 | 🞎 | 🞎 | 🞎 |
| GS5 | I am satisfied with family communication about my illness 0 | | 🞎 | 🞎 | 🞎 | 🞎 | 🞎 |
| GS6 | I feel close to my partner (or the person who is my main support) 0 | | 🞎 | 🞎 | 🞎 | 🞎 | 🞎 |
| Q1  GS7 | *Regardless of your current level of sexual activity, please* *answer the following question. If you prefer not to answer*  *it, please check this box and go to the next section.* | | |  |  |  |  |
|  | I am satisfied with my sex life………………………… | 🞎 | | 🞎 | 🞎 | 🞎 | 🞎 |

**QUALITY OF LIFE**

**By circling one (1) number per line, please indicate how true each statement has been for you during the past 7 days.**

|  | EMOTIONAL WELL-BEING | **Not at all** | | **A little bit** | **Some-what** | **Quite**  **a bit** | **Very much** |
| --- | --- | --- | --- | --- | --- | --- | --- |
|  |
|  |
|  |
| GE1 | I feel sad……………………………………………….. | | 🞎 | 🞎 | 🞎 | 🞎 | 🞎 |
| GE2 | I am satisfied with how I am coping with my illness….. | | 🞎 | 🞎 | 🞎 | 🞎 | 🞎 |
| GE3 | I am losing hope in the fight against my illness 0 | | 🞎 | 🞎 | 🞎 | 🞎 | 🞎 |
| GE4 | I feel nervous 0 | | 🞎 | 🞎 | 🞎 | 🞎 | 🞎 |
| GE5 | I worry about dying 0 | | 🞎 | 🞎 | 🞎 | 🞎 | 🞎 |
| GE6 | I worry that my condition will get worse 0 | | 🞎 | 🞎 | 🞎 | 🞎 | 🞎 |

|  | FUNCTIONAL WELL-BEING | **Not at all** | | **A little bit** | **Some-what** | **Quite**  **a bit** | **Very much** |
| --- | --- | --- | --- | --- | --- | --- | --- |
|  |
|  |
|  |
| GF1 | I am able to work (include work at home) 0 | | 🞎 | 🞎 | 🞎 | 🞎 | 🞎 |
| GF2 | My work (include work at home) is fulfilling 0 | | 🞎 | 🞎 | 🞎 | 🞎 | 🞎 |
| GF3 | I am able to enjoy life 0 | | 🞎 | 🞎 | 🞎 | 🞎 | 🞎 |
| GF4 | I have accepted my illness 0 | | 🞎 | 🞎 | 🞎 | 🞎 | 🞎 |
| GF5 | I am sleeping well 0 | | 🞎 | 🞎 | 🞎 | 🞎 | 🞎 |
| GF6 | I am enjoying the things I usually do for fun 0 | | 🞎 | 🞎 | 🞎 | 🞎 | 🞎 |
| GF7 | I am content with the quality of my life right now 0 | | 🞎 | 🞎 | 🞎 | 🞎 | 🞎 |

**QUALITY OF LIFE**

**By circling one (1) number per line, please indicate how true each statement has been for you during the past 7 days.**

|  | ADDITIONAL CONCERNS | **Not at all** | | **A little bit** | **Some-what** | **Quite**  **a bit** | **Very much** |
| --- | --- | --- | --- | --- | --- | --- | --- |
|  |
|  |
|  |
| B1 | I have been short of breath 0 | | 🞎 | 🞎 | 🞎 | 🞎 | 🞎 |
| B2 | I am self-conscious about the way I dress 0 | | 🞎 | 🞎 | 🞎 | 🞎 | 🞎 |
| B3 | One or both of my arms are swollen or tender 0 | | 🞎 | 🞎 | 🞎 | 🞎 | 🞎 |
| B4 | I feel sexually attractive 0 | | 🞎 | 🞎 | 🞎 | 🞎 | 🞎 |
| B5 | I am bothered by hair loss……………………………... | | 🞎 | 🞎 | 🞎 | 🞎 | 🞎 |
| B6 | I worry that other members of my family might someday get the same illness I have…………………… | | 🞎 | 🞎 | 🞎 | 🞎 | 🞎 |
| B7 | I worry about the effect of stress on my illness 0 | | 🞎 | 🞎 | 🞎 | 🞎 | 🞎 |
| B8 | I am bothered by a change in weight 0 | | 🞎 | 🞎 | 🞎 | 🞎 | 🞎 |
| B9 | I am able to feel like a woman 0 | | 🞎 | 🞎 | 🞎 | 🞎 | 🞎 |
| P2 | I have certain parts of my body where I experience significant pain 0 | | 🞎 | 🞎 | 🞎 | 🞎 | 🞎 |

**QUALITY OF LIFE**

By placing a tick in one box in each group below, please indicate which statements

Best describe your own health state today.

# Mobility

I have no problems in walking about ❏

I have some problems in walking about ❏

I am confined to bed ❏

# Self-care

I have no problems with self-care ❏

I have some problems washing or dressing myself ❏

I am unable to wash or dress myself ❏

# Usual activities *(e.g. work, study, housework, family or*

*leisure activities)*

I have no problems with performing my usual activities ❏

I have no problems with performing my usual activities ❏

I am unable to perform my usual activities ❏

# Pain/Discomfort

I have no pain or discomfort ❏

I have moderate pain or discomfort ❏

I have extreme pain or discomfort ❏

# Anxiety /Depression

I am not anxious or depressed ❏

I am moderately anxious or depressed ❏

I am extremely anxious or depressed ❏

| To help people say how good or bad a  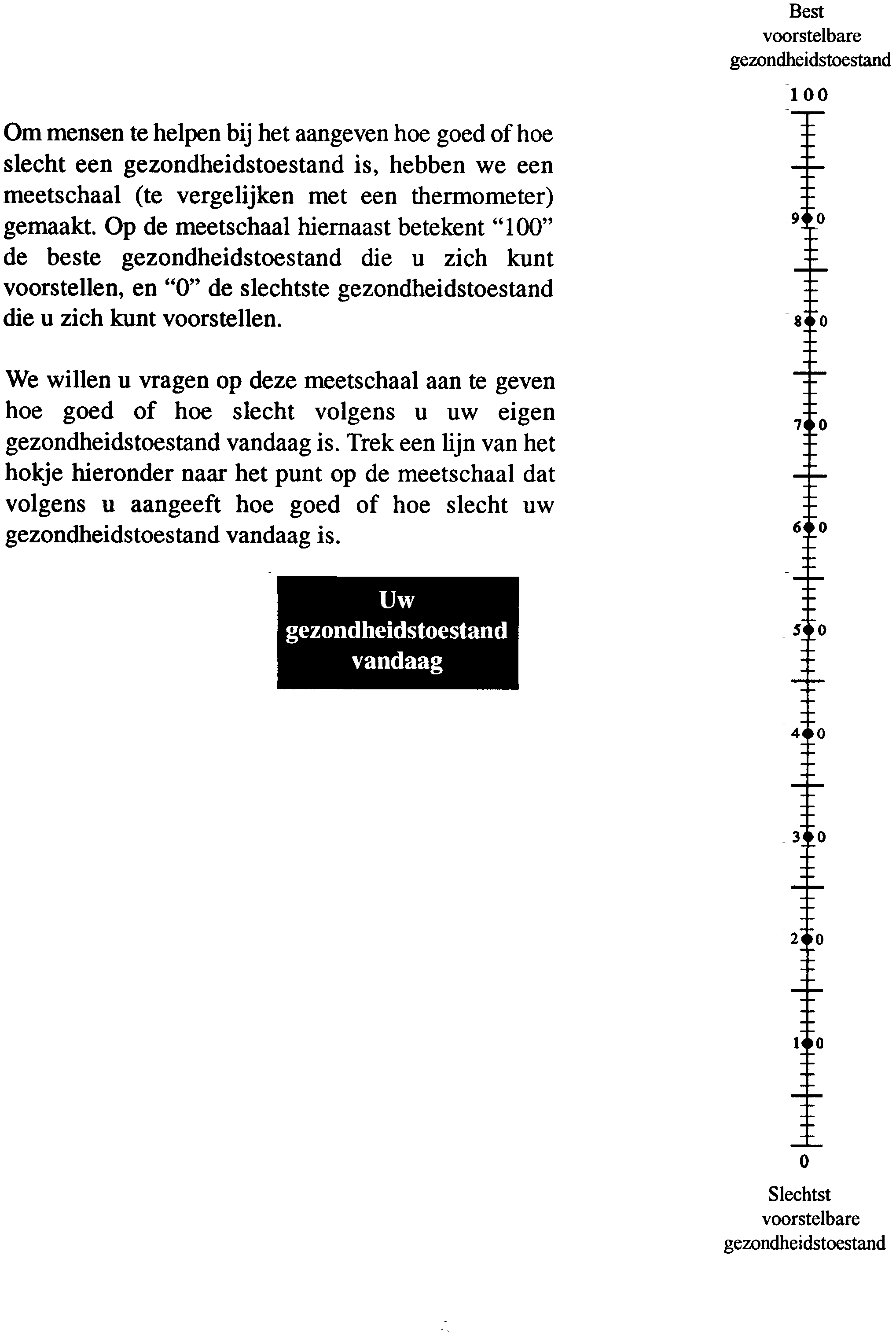health state is, we have drawn a scale  (rather like a thermometer) on which  the best health state you can imagine is  marked 100 and the worst state you can  imagine is marked 0.  Would you like to indicate on this  scale how good or bad your own health  is today, in your opinion. Please do  this by drawing a line from the box  below to whichever point on the scale  indicates how good or how bad your health  state is.  **Your own**  **health state**  Thank you very much for filling in this questionnaire! If you have any remarks, please tell us!  __________________________________  __________________________________  __________________________________  __________________________________  __________________________________ | Best imaginable  health state  Worst imaginable  health state |
| --- | --- |
